# Dietary total anti-oxidant capacity is inversely related to the prevalence of depression in adolescent girls

**DOI:** 10.1186/s12887-022-03589-4

**Published:** 2022-09-09

**Authors:** Tayebeh Zohrabi, Amirhosein Ziaee, Amin Salehi-Abargouei, Gordon A. Ferns, Majid Ghayour-Mobarhan, Sayyed Saeid Khayyatzadeh

**Affiliations:** 1 Nutrition and Food Security Research Center, Shahid Sadoughi University of Medical Sciences, Yazd, Iran; 2 Department of Nutrition, School of Public Health, Shahid Sadoughi University of Medical Sciences, Yazd, 8915173160 Iran; 3 Student Research Committee, Department of Nutrition, School of Medicine, Mashhad University of Medical Sciences, Mashhad, Iran; 4 Brighton & Sussex Medical School, Division of Medical Education, Falmer, Brighton, BN1 9PH Sussex UK; 5 International UNESCO center for Health-Related Basic Sciences and Human Nutrition, Mashhad University of Medical Sciences, Mashhad, Iran

**Keywords:** Antioxidants, Depression, Adolescent, Diet, Girl, Oxidative stress

## Abstract

**Background:**

Oxidative stress is considered to be a contributory factor for depression, and is affected by the dietary intake of pro-and anti-oxidants. Dietary total antioxidant capacity (DTAC) is an index which is applied to estimate the cumulative power of antioxidants in the whole diet. The aim of this study was to determine the relationship between DTAC and prevalence of depression in adolescent girls.

**Methods:**

A total of 741 Iranian adolescent girls aged 12–18 years were recruited into this cross-sectional study. Dietary intake and depression severity score were assessed using a food frequency questionnaire and Beck's depression inventory, respectively. To estimate the DTAC, the oxygen radical absorbance capacity method was used for selected foods. To explore the associations between DTAC and depression, logistic regression was applied using crude and adjusted models.

**Results:**

Individuals in the greatest adherence to high DTAC had more intakes of whole grains, legumes, fruits, dried fruits, low fat dairy products, cruciferous vegetables, fiber, magnesium, vitamin C, folate, potassium, zinc, β-carotene, lutein, thiamin, riboflavin, niacin and vitamin B-6 and lower consumption of refined grains. Subjects in the highest quartile of DTAC had a 39% lower odds of depression compared to those in the first quartile (OR = 0.61; 95% CI: 0.38–0.97, *P* for trend = 0.012); these associations remained significant after adjustments in first, second and third (OR = 0.5; 95% CI: 0.28–0.92, *P* for trend < 0.001) adjusted models.

**Conclusions:**

An inverse association was observed between the DTAC and the prevalence of depression in our population sample of adolescent girls. Further research needs to be conducted in different areas, including longitudinal studies with larger sample sizes.

## Introduction

Adolescents experience a high prevalence of depression, exhibit greater likelihood of attempted suicide, obesity, gastrointestinal dysfunction, type 2 diabetes, cardiovascular diseases and lower productivity, attention, sleep health and quality of life [1–3]. It is believed that by 2030, depression will be ranked among the three most common disorders responsible for global disease burden [4]. The prevalence of depression is estimated to be at least twofold in adolescent girls in comparison to other age and sex groups and hence these individuals are a vulnerable group for psychological disorders development [5]. Recently, the prevalence of depression was reported to be approximately 42.3% in Iranian children which was particularly high in high school girls [6]. Thus, the identification of modifiable risk factors of depression is an important research need, with a view to mitigating the diseases burden related to adverse depression outcomes. Oxidative stress is a major contributory factor for depression and other psychological disorders [7]. The imbalance between oxidative processes and antioxidant defenses leads to the oxidation of macromolecules such as proteins, lipids, and nucleic acids and cell membrane damage. When this balance is disturbed, oxidative stress is appeared as a consequence of overproduction of reactive oxygen species (ROS) and/or insufficiency of the antioxidant defense mechanisms and alter cellular functions [8]. Regarding to direct association between higher tissue oxygen consumption and greater production of free radicals in the brain, this organs is especially sensitive to oxidative damage [9]. Therefore, precious studies reported that oxidative stress plays a critical role in the pathogenesis of several disorders of our brain including neurodegenerative problems [10] and psychiatric illness [11].

Dietary antioxidants, such as polyphenols, vitamins A, C and E have the ability to scavenge free radicals, support the endogenous antioxidant system and protect against oxidative damages [12]. To calculate the antioxidant content of diets, dietary total antioxidant capacity (DTAC) has been introduced as an indicator that evaluates the cumulative power of antioxidant molecules in foods against reactive compounds [13]. An inverse relationship between DTAC with inflammation, oxidative stress and chronic diseases was reported in previous findings [14, 15]. DTAC was found as a protective factor for depression in two recent studies that performed in postmenopausal [16] and health adults populations [17]. However, little attention has been paid to the study of adolescents and we are not aware of any studies that have investigated the association between DTAC and depression in young people. Therefore, this cross-sectional study was performed in a sample of Iranian adolescent girls to examine the relationship between DTAC and depression.

## Material and methods

### Population

In this cross-sectional study, a total of 741 adolescent girls were recruited using a random cluster sampling method from several schools in different areas in the cities of Mashhad and Sabzevar, in northeastern Iran. Only students aged between 12 and 18 years without a history of chronic disease (colitis, diabetes, cardiovascular disease, cancer and hepatitis) were enrolled. All of the participants and their parents were asked to complete written informed consent before the study. The study protocol and tools were approved by Shahid Sadoughi University of Medical Sciences, Yazd, Iran, and ethics Committee (approval number, IR.SSU.SPH.REC.1400.131). All methods were performed in accordance with the ethical standards and with the relevant guidelines and regulations of the Declaration of Helsinki for Human participants.

### Demographic and clinical assessment

Demographic data including information on age (years), menstruation status, parent death, parent divorce (yes/no), educational level of parents (illiterate, primary, academic and advanced academic education) was collected at beginning of the study. Medical information including medical history, supplement and medication use and chronic diseases were obtained through standard questionnaire and by trained interviewers.

Physical activity information was obtained by using the validated Modifiable Activity Questionnaire (MAQ) [18]. Physical activity levels were computed based on metabolic equivalent task (MET) hours per week, (MET- h/week). Blood pressure was measured twice using a standardized protocol, and the average was recorded.

### Anthropometric measurements

Weight, height, hip and waist circumference (WC) were measured by using standard protocols and the mean of two measurements was reported. Body mass index (BMI) was calculated as weight (kg) divided by square of the height (m^2^). Waist to hip ratio was calculated by dividing WC by hip circumference. 

### Dietary assessment

Dietary intakes were evaluated using a validated and reliable food frequency questionnaire (FFQ) with 147 food items [19]. FFQs were completed during face-to-face interviews and reported dietary intakes in household measures were converted to grams and entered to the Nutritionist IV software. To calculate daily nutrient intake values for each participant, US Department of Agriculture’s (USDA) national nutrient databank was used [20]. The effect of energy intake was controlled by calculating the food group intakes per 1000 kcal.

### Dietary TAC calculation

DTAC was calculated using the Nutrient Data Laboratory of USDA Database based on the oxygen radical absorbance capacity (ORAC) method for selected foods, and expressed as μmol of Trolox Equivalents per 100 g of foods (μmol TE/100 g) [21].

### Depression evaluation

A Persian version of Beck's depression inventory (BDI) was used to determine depression status in the current study [22–24]. BDI is a self-administered questionnaire including 21 items with various options. The total score for the BDI ranges between 0 to 63 points. If the BDI score was < 13, the person was considered as not depressed, and if the subject’s score was > 13, they were categorized as depressed. The validity and reliability of BDI were assessed in previous studies (Cronbach’s α = 0.87 and acceptable test–retest reliability (r) = 0.74) [25–27].

### Statistical methods

All continuous variables were assessed for normality using the Kolmogorov–Smirnov test. Participants were categorized into quartiles of DTAC scores. Comparison of participant characteristics across the quartiles of DTAC was undertaken using one-way ANOVA for continuous variables (age, physical activity, weight, waist circumference, BMI, Depression score, Waist to hip ratio) and Chi-square test for categorical variables (BMI percentiles, passive smoking, menstruation, parental death, parental divorce, and educational attainment of parents). Crude and energy-adjusted dietary intakes of participants were compared across quartile categories of DTAC using one-way ANOVA. The association between DTAC and depression score was analyzed by Univariate and Multivariate Linear Regression. In addition, Multivariate logistic regression was used to find the relationship between adherence to DTAC and depression prevalence in several models. In first model, odds ratios (ORs) were adjusted for age and energy intake. Further adjustments were made for physical activity and menstruation. Finally, additional adjustment for BMI percentiles was performed in last model. All statistical analyses were performed using SPSS (Version 16.0; Chicago, IL), and *P* values < 0.05 was considered as a statistically significant level.

## Results

The characteristics of the study participants across quartiles of energy-adjusted DTAC are shown in (Table 1). No significant differences were found across quartiles of DTAC for All demographic and anthropometric variables. The individuals in highest quartiles of DTAC had lower depression score compared with those in the lowest quartiles (9.05 ± 8.1 vs. 12.1 ± 9.4; *P* = 0.01). Of the 741, 27.2% (*n* = 202) of study participants were depressed.

**Table 1:** General characteristics of study participants across quartiles of energy-adjusted dietary TAC

	**Quartiles of energy-adjusted dietary TAC**				
	**Q1 (**184**)**	**Q2 (**185**)**	**Q3 (**185**)**	**Q4 (**187**)**	*P*-value*
**TAC**	< 6926.7	6926.7- 8234.6	8234.7- 9784.9	> 9784.9	
**Age (Year)**	14.3 ± 1.5	14.4 ± 1.4	14.6 ± 1.5	14.6 ± 1.5	0.27
**Physical activity (MET. h/week)**	45.1 ± 3.44	45.4 ± 3.6	45.3 ± 3.4	45.4 ± 3.1	0.86
**Weight (Kg)**	52.4 ± 11.3	53.07 ± 11.47	53.3 ± 11.47	52.1 ± 12.83	0.75
**Waist circumference (cm)**	70.6 ± 8.92	70.5 ± 8.94	70.3 ± 8.8	70.3 ± 9.6	0.99
**BMI (kg/m**^**2**^**)**	21.6 ± 4.8	20.9 ± 3.8	20.9 ± 3.8	21.2 ± 4.2	0.28
**Depression score**	12.1 ± 9.4	11.3 ± 9.4	11.1 ± 9.7	9.05 ± 8.1	0.01
**Waist to hip ratio**	0.76 ± .05	0.76 ± .057	0.76 ± .056	0.77 ± .076	0.43
**BMI percentiles** (%)					
< 5	6.98%	6.55%	7.06%	4.30%	0.56
May-85	76.30%	81.90%	81.50%	77.70%	
85–95	11.29%	8.19%	9.28%	13.50%	
≥ 95	5.37%	3.27%	2.10%	4.30%	
**Passive smoker (%)**	20.50%	27.20%	23.30%	21.60%	0.26
**Menstruation (%)**	94.1	89.8	89.8	86.7	0.32
**Parent death (%)**	4.20%	2.60%	3.70%	4.70%	0.79
**Parent divorce (%)**	4.20%	3.70%	7.40%	3.70%	0.43
**Educational level of father**					
Illiterate	31.3	31.9	30.3	35.1	0.91
Primary	56.3	55.8	55.8	54.7	
Academic and advanced academic education	9.04	10.1	11.7	6.9	
**Educational level of mother**					
Illiterate	44.60%	42.50%	41.40%	42.50%	0.99
Primary	47.80%	47.80%	50.50%	48.90%	
Academic and advanced academic education	5.30%	6.90%	6.30%	6.30%	

*Abbreviations: DTAC* Dietary Total Anti-oxidant Capacity, *BMI* Body mass index, *MET. h/wk* Metabolic Equivalent Task. Hours/week, *Q* Quartile, *N* Number

^*^One-way ANOVA for continuous variables and Chi-squared test for categorical variables

The dietary intake for food groups of study participants are shown in (Table 2). Individuals with the highest adherence to DTAC, as would be expected, had higher intakes of whole grains, legumes, fruits, dried fruits, low fat dairy products and cruciferous vegetables and lower consumption of refined grains in crude and adjusted models.

**Table 2:** Dietary intakes of study participants by quartiles categories of dietary TAC

	**Quartiles of dietary TAC (N)**				
	**Q1 (184)**	**Q2 (185)**	**Q3 (185)**	**Q4 (187)**	*P*-value *
**DTAC**	< 6926.7	6926.7- 8234.6	8234.7- 9784.9	> 9784.9	
**Whole grains (g)**					
Crude	122.9 ± 118 ^I^	175.8 ± 146	248 ± 194	263 ± 194	< 0.001
Adjusted	64.08 ± 58	68.9 ± 53.2	82.7 ± 59.1	75.7 ± 51.3	0.007
**Refined grains (g)**					
Crude	282 ± 203	280 ± 185	299 ± 173	270 ± 136	0.43
Adjusted	143.1 ± 78	112.2 ± 61.7	102.7 ± 50.2	81.6 ± 42	< 0.001
**Legumes (g)**					
Crude	50.5 ± 36.8	72 ± 47.3	86.8 ± 60.1	124 ± 89.5	< 0.001
Adjusted	27.6 ± 22.6	30.4 ± 22.6	31.1 ± 24.2	37 ± 26.5	0.002
**Fruits (g)**					
Crude	115.3 ± 83	167.7 ± 111	216.8 ± 158	335.4 ± 240	< 0.001
Adjusted	61.1 ± 42	72.2 ± 60.4	77.8 ± 62.7	102.2 ± 73	< 0.001
**Dried fruits(g)**					
Crude	0.85 ± 1.6	2.02 ± 4	2.5 ± 5.5	5.9 ± 18.6	< 0.001
Adjusted	0.42 ± 0.7	0.7 ± 1.4	0.8 ± 1.6	1.7 ± 5.5	< 0.001
**Fruit juice(g)**					
Crude	10.7 ± 29.6	18.3 ± 42.3	27.5 ± 176	36.3 ± 95	0.09
Adjusted	5.1 ± 12	6.6 ± 13	8.1 ± 41.4	10.3 ± 24	0.24
**Fish(g)**					
Crude	6 ± 7.9	7.1 ± 9	8.9 ± 11.1	11.7 ± 23.8	0.001
Adjusted	3.2 ± 4.3	2.8 ± 3.4	3.1 ± 3.9	3.4 ± 6.4	0.64
**Low fat dairy(g)**					
Crude	117.5 ± 97.6	176 ± 148	248.7 ± 178	321.3 ± 269	< 0.001
Adjusted	63.3 ± 54	74.6 ± 65	88.5 ± 66.7	96.2 ± 78	< 0.001
**Green leafy vegetables(g)**					
Crude	9.3 ± 12.3	13.5 ± 23.4	14 ± 19	21.6 ± 24	< 0.001
Adjusted	4.8 ± 6.1	5.4 ± 8.4	4.7 ± 6.1	6.4 ± 7.5	0.09
**Cruciferous vegetables(g)**					
Crude	5.1 ± 9	6.4 ± 11.4	8.9 ± 17	15.6 ± 24.7	< 0.001
Adjusted	2.7 ± 4.7	2.7 ± 5.2	3.1 ± 6	4.6 ± 7.6	0.005
**Tomato(g)**					
Crude	45.4 ± 52.4	57.4 ± 58.8	70.4 ± 62.9	98.7 ± 112	< 0.001
Adjusted	25.4 ± 31.5	23.7 ± 23.9	24.8 ± 23.4	29.6 ± 33.8	0.19
**Nuts(g)**					
Crude	9.9 ± 26.9	15.7 ± 26.2	17.4 ± 24.3	22.4 ± 37.7	0.001
Adjusted	4.7 ± 9.4	6.2 ± 10.2	5.6 ± 6.7	6.1 ± 8.9	0.34

Values are expressed as mean ± SD

*Abbreviations: D TAC* Dietary Total Anti-oxidant Capacity, *Q* Quartile, *N* Number

^*^ Obtained from one-way ANOVA

The dietary intakes of fish, green leafy vegetables, tomato and nuts were significantly higher among individuals in the highest quartile for DTAC in crude model; but this did not remain significant after adjustment for energy intakes. There was no significant difference between dietary intakes of fruit juices across quartiles of DTAC in both models. Also, subjects in the lowest quartile of DTAC were more likely to consume higher intake of refined grains in energy-adjusted model.

The distributions of dietary nutrient intakes across the quartiles for DTAC are shown in (Table 3). Compared to those in the lowest adherence to DTAC, individuals in the highest quartile had significantly higher intakes of energy, fiber, magnesium, vitamin C, folate, potassium, β-Carotene, lutein, zinc, thiamin, riboflavin, niacin and vitamin B-6 in crude and energy adjusted models. A significantly higher consumption of vitamin A and vitamin E were seen among individuals with a greater adherence to DTAC, but only using the crude model. Also, there are no significant differences between dietary intakes of carbohydrate, protein and fat across quartiles of DTAC.

**Table 3:** Nutrient intakes of study participants according to quartiles of dietary TAC

	**Quartiles of dietary TAC (N)**				
	**Q1 (184)**	**Q2 (185)**	**Q3 (185)**	**Q4 (187)**	*P*-value*
**DTAC**	< 6926.7	6926.7- 8234.6	8234.7- 9784.9	> 9784.9	
**Dietary fiber (g)**					
Crude	35.65 ± 22	39.78 ± 17	49.37 ± 18	55.06 ± 18	< 0.001
Adjusted	18 ± 7.6	15.9 ± 5.6	16.8 ± 5.3	16.2 ± 4.7	0.006
**Magnesium (mg)**					
Crude	329.4 ± 122	447.5 ± 144	558.8 ± 171	663.3 ± 181	< 0.001
Adjusted	1697 ± 40	1798 ± 41	1898 ± 39	1946 ± 34	< 0.001
**Vitamin C (mg)**					
Crude	60.1 ± 35	83.3 ± 45	98.4 ± 52	140.7 ± 76	< 0.001
Adjusted	30.9 ± 16	34.6 ± 22	34.3 ± 19	42 ± 22	< 0.001
**Vitamin A (mcg)**					
Crude	4525 ± 919	5149 ± 277	6100 ± 303	8030 ± 422	< 0.001
Adjusted	2308 ± 436	2145 ± 127	2098 ± 94	2382 ± 119	0.6
**Folate (mcg)**					
Crude	464 ± 172	552 ± 161	657 ± 163	754 ± 179	< 0.001
Adjusted	239.7 ± 55	223.6 ± 49	226.3 ± 44	225.4 ± 48	0.007
**Potassium (mg)**					
Crude	2394 ± 728	3309 ± 844	4023 ± 852	5194 ± 1140	< 0.001
Adjusted	1242 ± 238	1341 ± 242	1386 ± 226	1545 ± 273	< 0.001
**β-Carotene (μg)**					
Crude	2164 ± 2048	2971 ± 2160	3442 ± 2263	4877 ± 3364	< 0.001
Adjusted	1124 ± 956	1242 ± 1006	1191 ± 759	1450 ± 969	0.005
**Lutein (μg)**					
Crude	1215 ± 899	1720 ± 1529	1916 ± 1458	2877 ± 2291	< 0.001
Adjusted	630.7 ± 436	701.9 ± 614	657.6 ± 476	850.6 ± 670	0.001
**Zinc (mg)**					
Crude	9.7 ± 3.8	12.7 ± 4.3	15.5 ± 4.7	18.1 ± 5.3	< 0.001
Adjusted	4.9 ± 1.1	5.1 ± 1.2	5.2 ± 1.03	5.3 ± .99	0.02
**Vitamin E (mg)**					
Crude	10.46 ± 6.4	12.57 ± 6.6	14.82 ± 5.6	16.64 ± 7.3	< 0.001
Adjusted	5.1 ± 2.4	5 ± 2	5 ± 1.6	4.8 ± 1.9	0.54
**Thiamin (mg)**					
Crude	1.7 ± .69	2.07 ± .71	2.54 ± .78	2.75 ± .79	< 0.001
Adjusted	.9 ± .19	.82 ± .18	.86 ± .17	.81 ± .17	< 0.001
**Riboflavin (mg)**					
Crude	1.5 ± .73	1.9 ± .53	2.4 ± .61	2.9 ± .9	< 0.001
Adjusted	.8 ± .3	.79 ± .19	.83 ± .17	.86 ± .23	0.007
**Niacin (mg)**					
Crude	19 ± 7.3	22.3 ± 8.3	26.9 ± 8.6	28.9 ± 8.3	< 0.001
Adjusted	9.7 ± 2.1	8.8 ± 1.9	9.1 ± 1.9	8.5 ± 1.8	< 0.001
**Vitamin B-6 (mg)**					
Crude	1.3 ± .44	1.7 ± .49	2 ± .49	2.4 ± .58	0
Adjusted	.7 ± .14	.69 ± .12	.7 ± .11	.73 ± .13	0.02
**Monounsaturated fat**					
Crude	24.07 ± 12.2	31.6 ± 13.1	35.6 ± 12.7	40.9 ± 14.8	0
Adjusted	11.9 ± 3.8	12.5 ± 3.4	12.1 ± 3.3	11.8 ± 3.1	0.22
**Polyunsaturated fat**					
Crude	17.07 ± 9.8	21.9 ± 11.1	24.8 ± 10.8	27.7 ± 12.9	0
Adjusted	8.3 ± 3.2	8.6 ± 3.2	8.4 ± 3.1	8.0 ± 2.9	0.24
**Saturated fatty acids**					
Crude	30.6 ± 14	32.04 ± 14	30.3 ± 11.7	28.6 ± 13.4	0.91
Adjusted	10.6 ± 3.6	11.5 ± 3.3	11.3 ± 3.1	11.2 ± 3.3	1.17
**Total energy (Kcal)**	1976 ± 642	2508 ± 640	2944 ± 645	3400 ± 648	< 0.001
**Carbohydrate(% of total energy)**	.55 ± .08	.54 ± .06	.55 ± .06	.55 ± .07	0.38
**Protein (% of total energy)**	.135 ± .02	.133 ± .02	.138 ± .02	.138 ± .02	0.05
**Fat (% of total energy)**	.33 ± .08	.34 ± .06	.33 ± .06	.33 ± .07	0.16

Values are expressed as mean ± SD

*Abbreviations: DTAC* Dietary Total Anti-oxidant Capacity, *Q* Quartile, *N* Number

^*^ One-way ANOVA was used to test dietary intake across the quartiles of dietary TAC

Multivariate adjusted odds ratios and 95% confidence intervals (CIs) for the likelihood of depression across quartiles of DTAC are shown in (Table 4). Using an unadjusted model, the subjects in the highest quartile of DTAC had a 39% lower probability of having depressive symptoms compared to those in the first quartile (OR: 0.61; 95% CI: 0.38–0.97, *P* for trend = 0.01). After controlling for age and energy intake in the first model, individuals in the highest quartile of the DTAC were 50% less likely to experience depression symptoms compared to those in the lowest quartile (OR = 0.50; 95% CI: 0.28–0.90, *P* for trend < 0.001). Further adjustments for physical activity and menstruation were done in model II. The OR for participants in the highest, compared with the lowest quartile of DTAC was 0.51; 95% CI: 0.28–0.93 and *P* for trend < 0.001 in second model. After final adjustment for BMI percentile, these findings remained significant in last model (OR = 0.50; 95% CI: 0.28–0.92, *P* for trend < 0.001).

**Table 4:** Multivariable- adjusted odds ratio (95% CIs) for depression across quartiles of dietary TAC

	**Quartiles of dietary TAC (N)**				
	**Q1 (184)**	**Q2 (185)**	**Q3(185)**	**Q4 (187)**	*P* trend*
**DTAC**	< 6926.7	6926.7- 8234.6	8234.7- 9784.9	> 9784.9	
**Crude**	1.00	0.96 (0.62–1.5)	0.64 (0.4–1.01)	0.61 (0.38–0.97)	0.01
**Model I**	1.00	0.90 (0.56–1.42)	0.50 (0.33–0.94)	0.50 (0.28–0.9)	< 0.001
**Model II**	1.00	0.9 (0.57–1.43)	0.57 (0.33–0.97)	0.51 (0.28–0.93)	< 0.001
**Model III**	1.00	0.85 (0.53–1.36)	0.55 (0.32–0.94)	0.50 (0.28–0.92)	< 0.001

Model I: adjusted for age and energy intake; Model II: additionally, adjusted for physical activity and menstruation; Model III: further adjustments for BMI percentile

*Abbreviations: DTAC* Dietary Total Anti-oxidant Capacity, *Q* Quartile, *N* Number

^*^ *P* for trend based on logistic regression

The association between DTAC and depression score is shown in Table 5. There was an inverse correlation between DTAC and depression score in unadjusted model (β standardized = -0.107; *P* value = 0.003). Likewise, this inverse relationship was significant after adjustment for confounding factors in Model I (β standardized = -0.114; *P* value = 0.002), Model II (β standardized = -0.108; *P* value = 0.005), Model III (β standardized = -0.108; *P* value = 0.005).

**Table 5:** The association between dietary TAC and depression score

	**ß un standardized**	**ß standardized**	*P*-value*	**95% Confidence Interval**
Depression score				
Crude	-3.94	-0.107	0.003	-6.58, -1.3
Model I	-4.17	-0.114	0.002	-6.87, -1.47
Model II	-3.96	-0.108	0.005	-6.70,-1.23
Model III	-3.97	-0.108	0.005	-6.71,-1.23

Model I: adjusted for age and energy intake; Model II: additionally, adjusted for physical activity and menstruation; Model III: further adjustments for BMI percentile

*Abbreviations: TAC* Total Anti-oxidant Capacity, *ß* Beta

^*^ Univariate and Multivariate regression were used to test the association between dietary TAC and depression score in crude and adjusted model, respectively

## Discussion

The results of our cross-sectional study showed that a higher total antioxidant capacity is associated with lower prevalence of depression in adolescent girls. An inverse correlation was found between DTAC and depression score. Oxidative stress is proposed as one of the potential mechanisms in pathophysiology of depression [28] and previous reports indicated which diet might have a regulatory role in oxidative stress [29]. Recently, DTAC has been used as a novel indicator for assessment of diet quality that is associated with serum oxidant-antioxidant balance [13]. Although several studies have examined the role of dietary factors in mental disorders [30], little is known about the association between dietary TAC and psychological conditions among adolescent girls, while are vulnerable group for the development of mental disorders. To the best of our knowledge, this is the first study designed to evaluate the relationship between DTAC and depression in adolescent girls.

In this study, the individuals with higher scores for DTAC consumed greater amounts of whole grains, legumes, fruits, dried fruits, cruciferous vegetable and low fat dairy whereas there are lower intakes of refined grains, previous studies have also shown that the adults with symptoms of depression consume fewer vegetables, fruits and grains and have lower DTAC [17, 31]. According to the results of meta-analysis, consumption of fruits and vegetables were inversely related to the risk of depression [32]. Fruits, vegetables and whole grain are known as mains sources of dietary antioxidants [33] and reduced risk of psychotic problems were seen in populations that consumed more amount of high rich antioxidant food [34]. These results are consistent with the findings of our study that whole grains, fruits and vegetables, in particular cruciferous vegetable, were more associated with higher DTAC.

A greater adherence to a diet rich in DTAC was associated with a lower odds of depression in adolescent girls in our study and this finding was in line with those from earlier studies that reported the consumption of foods with presumed anti-inflammatory properties, e.g. beans, fruits and vegetables, whole grain, fish, olive oil, low-fat dairy and antioxidants (e.g. vitamin C, vitamin E, Vitamin B6, zinc, flavonoids, and carotenoids) may lower risk of depression [30, 35]. In a cross-sectional study in a large sample of Iranian adults, a strong positive association was found between pro-inflammatory dietary index and psychological problems especially in women [36] and similar results were obtained among Iranian adolescent girls [37]. According to the dietary inflammatory index (DII), fiber, magnesium, zinc, vitamin A, vitamin C, vitamin E, vitamin D, vitamin B6, folic acid, beta-carotene, tea, turmeric, garlic and onions were defined as anti-inflammatory components [38] that may support our findings that individuals in highest quartiles of dietary TAC received more amounts of dietary fiber, magnesium, vitamin C, folate, zinc, β-carotene, thiamin, riboflavin, niacin and vitamin B-6.

The mechanisms through which the total anti-oxidant capacity might contribute to the etiology of depression are not well understood, but it is well established that oxidative-stress has an important role in the etiology of depression [39]. The anti-inflammatory properties of DTAC components were proposed as one of the principal pathways that might reduce risk of psychological disorders. In this regard, higher adherence to DTAC potentiate antioxidant levels, while lower free radical and oxidative stress in depressed patients [40]. Elevated serum concentrations of pro-inflammatory markers such as, C- reactive protein are present in depressive patients [41]. There is evidence of a positive association between intestinal permeability and the severity of depressive symptoms. A possible mechanism that links immune activation to the onset of depressive symptoms may be related to the neurotoxic properties of oxidative stress [3]. The connection between the gut and the brain includes neural, metabolic, endocrine and immunological pathways. Stress causes the hypothalamic neurons to secrete corticotrophic receptor hormone, and stimulate the release of adrenocorticotrophic releasing hormone, and subsequently cortisol, which affects intestinal barrier integrity and health. This leads to increased intestinal and blood brain barrier permeability, resulting in increased peripheral immune responses, and increased oxidative stress in central nervous system [42]. A diet characterized by high intake of refined sugars, animal fats, processed meats, refined grains, high-fat dairy products, can impair intestinal barrier function. This increases intestinal permeability and leakage of toxic bacterial metabolites into the circulation, and also leads to low-grade inflammation [43]. There is emerging evidence evaluating the role of gender in the gut-brain axis [44].Previous research has shown gender differences in cortisol response [45]. Cortisol response to stress may be associated with increased risk of unhealthy eating behaviors in adolescents [46] and may mediate the association between depression and anthropometric parameter (BMI) in girls [45]. In this study, we examined more diverse determinants of depression such as anthropometric parameters, physical activity [45], age, parents’ educational level and other social determinants [47], factors mentioned in Table 1. Comparison of these variables both in the DTAC classification and between people with depression and people who did not experience depression did not show any significant difference. Therefore, the effect of these variables has somehow been minimized in our study. Despite all this; Variables were included as confounders in our models to double control for potential effects and actually capture the results.

Another possible mechanism of TAC is associated to protective effects of anti-oxidant rich diet on chronic diseases. Metabolic complications are considered as major risk factors for psychological disorders, and previous epidemiological studies suggested greater adherence to DTAC reduces the risk of depression- related chronic diseases including diabetes mellitus, metabolic syndrome [14, 48, 49]. A diet high in glycemic and saturated lipid load predisposes the brain to oxidative stress and leads to insulin resistance, which is associated with more depressive symptoms, general mood disorders and fatigue [50, 51]. Fatigue is one of the most common symptoms of depression, affecting more than 90% of patients, and is associated with irritability, poor concentration, and decreased productivity [52, 53].

This study was conducted in a large sample and is one of the first studies to examine the relationship between DTAC and prevalence of depression in adolescent girls. To avoid misleading conclusions in analysis and interpretation of data, we conducted several adjustment models for confounding factors to depression. Nevertheless, our findings should be interpreted in the light of some potential limitations. Cross-sectional design should be considered as the major limitation of the present study; and cannot therefore assume a causal relationship. Plasma total antioxidant capacity was not measured in the present study. To assess dietary intake, we used FFQ method. FFQs are prone to measurement error and misclassification. Also, we did not examine the specific symptoms of depression, (such as appetite, fatigue), and its association with DTAC quartiles, but it is important to be considered in future research. Finally, like other observational studies, several unmeasured confounders were in this study, which we are unable to control them.

## Conclusion

In summary, our findings demonstrated that a high DTAC is associated with lower prevalence of depression in adolescent girls. The more adherences to healthy dietary pattern high in antioxidant might reduce oxidative stress and improve psychological health and productivity among children. In addition, adolescent-centered nutritional interventions should be designed as a school snack with a greater emphasis on fortifying foods with fruits and vegetables. To confirm these findings, further research needs to be conducted in different areas, including longitudinal studies with larger sample sizes.

## Data Availability

The data and materials of the current study is available from the corresponding author on reasonable request.

## Abbreviations

DTAC: Dietary Total Anti-Oxidant Capacity
FFQ: Food frequency questionnaire
BDI: Beck's depression inventory
MAQ: Modifiable Activity Questionnaire
MET: Metabolic equivalent task
ORs: Odds ratios
CIs: Confidence intervals
ORAC: Oxygen radical absorbance capacity
DII: Dietary inflammatory index
BMI: Body mass index
WC: Waist circumference
